# LCA of 1,4-Butanediol Produced via Direct Fermentation of Sugars from Wheat Straw Feedstock within a Territorial Biorefinery

**DOI:** 10.3390/ma9070563

**Published:** 2016-07-12

**Authors:** Annachiara Forte, Amalia Zucaro, Riccardo Basosi, Angelo Fierro

**Affiliations:** 1 Dipartimento di Biologia, Università di Napoli Federico II, Napoli 80126, Italy; amalia.zucaro@unina.it (A.Z.); fierro@unina.it (A.F.); 2 Dipartimento di Biotecnologie, Chimica e Farmacia, Università di Siena, Siena 53100, Italy; riccardo.basosi@unisi.it; 3 Laboratorio di Urbanistica e di Pianificazione del Territorio (LUPT), Università di Napoli Federico II, Napoli 80138, Italy

**Keywords:** 1,4-butanediol, LCA, bio-based chemicals, renewable feedstocks, biotechnology, climate change, environmental impacts

## Abstract

The bio-based industrial sector has been recognized by the European Union as a priority area toward sustainability, however, the environmental profile of bio-based products needs to be further addressed. This study investigated, through the Life Cycle Assessment (LCA) approach, the environmental performance of bio-based 1,4-butanediol (BDO) produced via direct fermentation of sugars from wheat straw, within a hypothetical regional biorefinery (Campania Region, Southern Italy). The aim was: (i) to identify the hotspots along the production chain; and (ii) to assess the potential environmental benefits of this bio-based polymer versus the reference conventional product (fossil-based BDO). Results identified the prevailing contribution to the total environmental load of bio-based BDO in the feedstock production and in the heat requirement at the biorefinery plant. The modeled industrial bio-based BDO supply chain, showed a general reduction of the environmental impacts compared to the fossil-based BDO. The lowest benefits were gained in terms of acidification and eutrophication, due to the environmental load of the crop phase for feedstock cultivation.

## 1. Introduction

In the last decades global policy decisions have been orienting toward sustainable strategies aiming to: (i) reduce fossil fuel dependency and linked environmental impacts; and (ii) generate new economy. The issue of sustainable consumption is complex and requires composite mix of different implementation policy instruments to address the different involved stakeholders [1,2]. Accordingly, the European Union (EU) policy encourages research activities and policies in order to reach: (i) the reduction of overconsumption pattern driven by lifestyle [2,3,4,5,6]; and (ii) cleaner production chains, strengthened by the EU Action Plan on Sustainable Consumption and Production and Sustainable Industrial Policy [7]. Specifically, as it relates to the latter key point, i.e., “the sustainable products”, the EU has recognized the bio-based sector as a priority area with high potential for: Sustainable economy (lower dependency on fossil fuels), future growth, re-industrialization and societal challenges [8]. Industrial sectors are increasingly involved in developing environmentally-friendly supply chains from renewable feedstocks, through substitution and energetic implementation of traditional petroleum-based routes still dominating our overall standard of living [9,10,11,12,13]. To this end, the bio-based materials, entirely or partlyproduced from biological feedstocks, such as food crops, wood, grass and agricultural by-products, may represent a significant and growing market with an extensive range of products. In this context, the scientific research plays a pivotal role in improving technical processes, but, above all, the proper knowledge in evaluating the sustainability of the complex system, represented by the bio-refinery.

The 1,4-Butanediol (BDO) is an organic compound commonly used as a solvent in the industrial cleaners and in the annual manufacture of over 2.5 million tons of valuable polymers [10]. It represents a key chemical building block used to make several products, such as polybutylene terephthalate (PBT), spandex (lycra, elastane), and polyurethanes (e.g., car bumpers). It was first commercially produced in 1930, through the reaction of formaldehyde with acetylene and subsequent stages of hydrogenation (Reppe chemistry). Nowadays, in the petrochemical industry, BDO can be produced also in different ways from maleic anhydride, propylene oxide and butadiene [10,14]; however the Reppe process still accounts for about 42% of global production [14].

The bio-based BDO manufacture process is not performed worldwide on large scale yet. It can either take place via hydrogenation of succinic acid [15,16] or direct fermentation of sugars through experimental metabolic engineered bacterial platform [10,12]. The latter one represents an emerging technology, which is claimed as an effective advantageous alternative over the succinic acid route, with a lower risk of market fragmentation and competition, thanks to the use of more abundant feedstock [14]. Several EU and worldwide companies are currently developing and up-scaling bio-based BDO production, also through direct fermentation of vegetal biomass sugars [10,12,14], on the basis of the generally claimed less energy and emission intensive pattern in comparison to petroleum-based counterparts.

The actual pertinence of the innovative bio-based technologies, framed on feedstock different than fossil oil, needs multi-criteria studies since it causes radical environmental, industrial and social changes. Regarding the environmental impacts of such a new bio-based chemical products, the life cycle assessment (LCA) represents an effective analytical tool [13,17,18]. All the direct and indirect environmental impacts associated with the product, process or activity are included in the assessment. This one encompasses the extraction and processing of raw materials, manufacturing and assembly processes, product distribution, use, maintenance, recycling and disposal [19,20]. Previous publications have outlined the effect of green chemistry on several aspects of a product’s life-cycle, using the LCA approach to assess the trade-offs in switching between supply chemicals or processes [16,18,21,22,23,24]. On one hand, bio-based building blocks appeared to entail relevant benefits in terms of non-renewable fossil energy saving and global warming mitigation potential [11,16,18,25,26,27]. On the other hand, contrasting findings arose while investigating the whole environmental performance of bio-based polymers [18,28,29].

Considering the innovative nature of bio-based BDO, there is still a lack of comprehensive analyses about the environmental profile of its production chain. Specifically, no published studies quantitatively assessed the effect of the production of BDO via direct fermentation of renewable sugars from lignocellulosic feedstock. In this regard, the present study aims to assess the environmental load of bio-based BDO, in order to evaluate potential improvements and benefits compared to the fossil counterpart. The inventory was based on experimental primary data (inherent the fermentation and purification steps) completed by secondary data for upstream lignocellulosic feedstock pre-treatment/hydrolysis and regional agronomic supply chains, assuming the energetic valorization of unconverted solids through combustion in a combined heat and power cogeneration system (CHP) located inside the biorefinery plant.

## 2. Materials and Methods

The methodological framework used in this paper was the LCA as defined by ISO standards and ILCD Handbook guidelines [19,20,30,31]. This internationally accepted method provides quantitative, confirmable, and manageable process models to evaluate production processes analyze options for innovation and improve understanding of the investigated systems [32]. An LCA analysis includes four primary stages: (1) definition of the goal and scope (including the selected functional unit and investigated system boundaries); (2) inventory analysis, where input and output flows are quantified along the investigated supply chain; (3) impact analysis, to determine the potential environment loads; and (4) interpretation of results, which include the implementation of optimization procedures in order to reduce impacts of the investigated product or process.

### 2.1. Goal and Scope

An attributionalcradle-to-factory gate LCAwas applied to a hypothetical local biorefinery scenario in Campania Region (Southern Italy) for the production of bio-based BDO via direct fermentation of sugars from wheat straw feedstock.

The aim of the study was to: (i) assess the environmental load of bio-based BDO, quantifying the resources use and emissions along the production chain; (ii) evaluate opportunities for improvements of the bio-product supply chain; and (iii) compare the environmental performance of bio-based BDO with fossil-based counterpart, in order to ascertain the potential environmental benefits of this foreseen eco-friendly bio-polymer.

**Figure 1:**
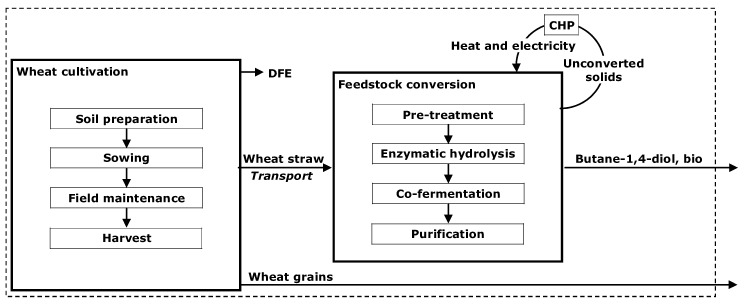
Analyzed production stages of bio-based BDO production chain. The dotted frame shows the “cradle-to-factory gate” system boundary investigated in the study.

To this end, according to the LCA procedures, data were collected considering the different production stages inside the “cradle-to-factory gate” system boundary (Figure 1).

The primary data about the bio-based BDO production via direct fermentation of sugars were gathered within the framework of the “EnerbioChem” project (fermentation and purification stages). Differently, a review of pertinent technical and scientific literature was carried out to: (i) check the local availability of wheat straw feedstock; (ii) identify the common agronomic practices for wheat cultivation in the context of Campania Region; and (iii) retrieve material and energetic input for the industrial recovery of fermentable sugars from lignocellulosic feedstock (pre-treatment and enzymatic hydrolysis) and the energetic valorization of unconverted solids inside the designed biorefinery system (electricity and heat production through combustion in a cogeneration plant).

Collected data were analyzed according to ISO standards [19,20], by means of SimaPro 8.0.3 software [33] coupled with the ReCiPe (v.1.10, 2013) [34] midpoint hierarchic impact assessment method.

For the wheat cultivation, the study included the co-production of wheat grains (sold to the market) and wheat straw (used into the investigated hypothetical regional bio-refinery chain) (see Section 2.2.1 for more details). For this reason, as suggested by ISO standards [19,20] and ILCD [30,31], the impacts of wheat cultivation were allocated on economic basis between the two co-products, on the basis of 10-years averaged prices for grains and straws of durum and common wheat (statistics downloaded from regional chamber of commerce [35,36]): 85% to grains and 15% to straw. The raw materials, energy use, manufacturing processes, transport, distribution, use and the final disposal of auxiliary inputs within and between each production stages were considered. Otherwise, the study did not encompass: (i) the use phase (BDO can be used in multiple products, each one consumed at different rates) and (ii) the disposal phase (the environmental impacts of bio-polymer disposal have not yet been studied extensively and there is a lack of adequate data on emissions, energy use and inherent degradation) [18]. In this regard, the biogenic C incorporated within the bio-based BDO molecule was considered as carbon-neutral. Otherwise, no CO_2_ uptake was included in the present analysis, since the long term storage of biogenic C inside bio-based compounds is highly affected by the final use and durability of the target biopolymers.

In order to address advantages or disadvantages of the investigated bio-based BDO production chain and allow suitable comparison with other bio-based or fossil-based products, the functional unit (FU) was set as 1 kg of bio-based BDO.

An uncertainty analysis and a sensitivity check were also carried out to assess how the final outcome of the study might be affected by assumptions on foreground input/output data and by alternative biomass feedstock/residues processing routes.

### 2.2. Life Cycle Inventory (LCI)

As far as the fossil-based BDO was concerned, inventory data were retrieved from the EcoInvent database v. 2.0 [37]: record “butane-1,4-diol, at plant”.

As regards the bio-based BDO, the inventory analysis (detailed in Section 2.2.1 and Section 2.2.2) quantified flows input (using mass and energy balances) and output (products and pollutant emissions released to air, water and soil) for all the processing steps included in the system boundary (Figure 1).

#### 2.2.1. LignocellulosicFeedstock Cultivation

For this analysis, we considered wheat straw as eligible lignocellulosic feedstock for the BDO supply chain. According to the Italian National Agency for New Technologies, Energy and Sustainable Economic Development [38], large areas of wheat crops are grown in Campania Region (about 120 kha, i.e., 12% of the total regional agronomic surface), entailing about 210 kton·y^−1^ of straw by product. Complying with the regional technical specifications [39], cereal stubble can be: (i) removed from the field, in case of inoculum of pathogens diseases and/or for on-farm/off-farm use as livestock feed and bedding; or (ii) incorporated in soil through disk harrowing (after cutting). In the latter case, 5–10 kg of additional N fertilizer need to be supplied per ton of incorporated straw, in order to reduce C/N ratio. Following ENEA estimates [38], about 70% of total produced straw would be used for agronomic and zootechnical purposes, whilst the remaining, about 55 kton·y^−1^, might be further valorized inside integrated bio-energy chains. Indeed, in Campania Region stalks are often seen as a waste disposal problem. As far as wheat straw are concerned, farmers are sometimes discouraged to both permitted routes, due to: (i) time and money expensive operations for biomass baling and transport, above all under the off-farm use as output for sale (in case of straw removal); and (ii) delayed establishment of the following crop, additional chopping operations and N additional fertilizer input (in case of straw incorporation in soil). As a result, the burning of cereal stubble, prohibited by the regional regulations [39], is still a potential relevant cause of brush fire in the regional context [40].

In this study, only the actual available wheat straw (WS) biomass in Campania Region (not used for agronomic and zootechnical purposes), was considered as potential feedstock for a hypothetical bio-based manufacturing process of BDO.

Table 1 shows the best guess (BG) value (the best estimated value used in this study) and the uncertaintyrange for each agronomic foreground input and output related to wheat cultivation in Campania Region, as retrieved from regional technical specifications [39], statistics [38] and pertinent scientific literature [41,42,43,44,45,46,47,48,49,50,51]. In detail, specific agronomic input data were collected: from the site preparation until the harvest operation, considering type of agricultural machineries, seeding operations, type and application rate of mineral fertilizers and the other auxiliary chemicals. Afterwards, starting from the EcoInvent (v. 2.0) database [37], specific records were implemented for all the agricultural practices through the SimaPro software [33], in compliance with the reviewed pertinent literature and agronomic technical specifications for the analyzed regional context [38,39,40,41,42,43,44,45,46,47,48,49,50,51].

Direct field emissions (DFE) of reactive nitrogen (volatilized ammonia, biogenic dinitrogen monoxides, nitrogen oxides, leached nitrate), carbon dioxide (fossil, from urea molecule decomposition in soil) and P losses (phosphorus/phosphate runoff to surface water and phosphate leaching to ground water, in case of additional P-K synthetic fertilizers) were computed according to EcoInvent guidelines [47,48] and IPCC methodology [48], through the use, when possible, of pedo-climatic and site-crop specific data, relative to wheat cultivation in the Mediterranean context [46].

The direct biogenic N_2_O emissions, following fertilizer application, were calculated over an average estimate of emission factor (EF) for spring-summer Mediterranean crops (0.5%) [46]. This value is markedly lower than the default EF (percentage of kg N-N_2_O per kg N-fertilizer input applied) of 1% considered by IPCC [48].

#### 2.2.2. Feedstock Conversion in the Industrial Plant

The study assumed a regional short-distance supply scenario of the lignocellulosic feedstock to the biorefinery plant (70 km), (DM 2/03/2010).

The average wheat straw composition was assumed as follow: 37% cellulose, 28% hemicellulose, 20% lignin, 5% ashes, 10% other organics and extractives [52,53,54].

Energy and material inputs for the pre-treatment (dilute acid) and saccharification (enzymatic hydrolysis) were secondary data retrieved from the EcoInvent database v. 2.02 and guidelines [55] and pertinent scientific literature [23]. According to Volynets and Dahman [53] an average 74% recovery efficiency of total sugars after pre-treatment and saccharification was assumed. The preliminary primary data for energy and material (nutrients and chemicals) inputs along the fermentation and purification steps were available inside the framework of the EnerbioChem Project. The BDO product purity is a crucial parameter to meet the current commercial requirements for polymer-based supply chains. High purity BDO was achieved through standard industrial recovery technologies, according to the following steps: cell and salt separation, water evaporation (to be further recycled within the system) and final BDO purification. Inputs and process efficiency at these stages represent sensitive industrial data. For this reason, all the industrial processing data (foreground and background) were disclosed aggregated (Table 2). The entire outcome of the LCA analysis was referred to an averaged medium scenario of co-fermentation efficiency of cellulose and hemicellulose equal to 80% of the maximum stechiometric bio-based BDO yield. Additionally other two fermentation efficiency scenarios were considered and encompassed inside the study: 100% (best-case) and 70% (worst-case) of the maximum stechiometric yield.

Within the designed bio-refinery system, the recovered C6 and C5 sugars were used for the co-fermentation process, whilst (ii) the lignin residues, the not hydrolyzed holocelluloses and the not fermented sugars were assumed to be recovered as unconverted solids (US) and burned beneficially, in a cogeneration unit system (Combined Heating and Power plant, CHP) for the on-site generation of process heat and electricity (Figure 1).

Table 2 summarizes the input flows related to the whole feedstock conversion process from wheat straw towards bio-based BDO (data input and output are referred to the selected FU: 1 kg of BDO).

According to the best estimated value (BG) for the unconverted solids characteristics, related to the default averaged medium scenario of co-fermentation efficiency (summarized in Table 3), the system might be self sufficient as far as the electricity input is concerned; whilst an additional amount of heat (assumed supplied by natural gas burning) would be necessary. Following the EcoInvent guidelines [55], the input and output pollutant emissions related to the CHP plant were calculated by adapting the record “wood chips, burned in cogen 6400 kWth, emission control” on the basis of dry matter, carbon and energy content of the analyzed unconverted solids.

The effect of the different fermentation efficiencies on the total amount of input process and available unconverted solids to CHP plant (and the system energy balance) was also taken into account. For this reason, the uncertainty ranges of industrial input and output were derived respecting the three different fermentation scenario efficiencies: (i) on a linear basis for material industrial input (water, chemicals and nutrients), according to the different wheat straw biomass input required; and (ii) in compliance with the different modeled US amount and characteristics, for energy balance and pollutant emissions from the CHP (Table 3).

The background inventory data (i.e., extraction and treatment of raw materials, manufacturing process, transport, distribution, use phase and the final disposal) for machineries and infrastructures were retrieved from the EcoInvent product or process database (v. 2.02) [37].

### 2.3. Life Cycle Impact Assessment (LCIA)

LCIA phase assigns qualitative and quantitative environmental effects to all foreground and background data collected during the inventory analysis. Environmental impacts are computed based on the selected functional units.

In this study the LCIA was performed by means of the LCA software SimaPro 8.0.3 [33] and one of the most recent and up-to-date LCA methods, Europe ReCiPe H v. 1.10 midpoint method [34]. The ReCiPe method can be profitably used to assess environmental impacts for a large number of upstream (i.e., referred to depletion of natural resources, such as fossil and water depletion categories) and downstream (i.e., referred to impacts generated on the natural matrices, such as terrestrial, marine or freshwater acidification) impact categories [34]. The midpoint level was chosen in this study, since it characterizes impacts somewhere in the middle of the environmental cause-effect chain and it is widely recognized as more robust and less uncertain compared with endpoint indicators [34]. The ReCiPe method provides characterization factors to quantify the contribution of processes to each impact category and normalization factors to allow a comparison across categories [34]. The normalization step is not mandatory in the ISO 14040-14044 standards [19,20], nor it is in the ILCD Handbook [30]. The main reason is that this step is still very uncertain and based on parameters considered arbitrary. As a consequence, the interpretation phase (result and discussion session) in this study was based on the characterization diagrams and the absolute value of impacts focusing on their breakdown into the different impact sources.

The following impact categories were explored in this work: Climate Change (CC, kg CO_2_ eq), Ozone Depletion (OD, kg CFC-11 eq), Terrestrial Acidification (TA, kg SO_2_ eq), Freshwater Eutrophication (FE, kg P eq), Marine Eutrophication (ME, kg N eq), Photochemical Oxidant Formation (POF, kg NMVOC), Particulate Matter Formation (PMF, kg PM10 eq), Water Depletion (WD, m^3^) and Fossil Depletion (FD, kg oil eq).

The toxicity impact categories were not evaluated, due to the large uncertainty still affecting their computation [57].

### 2.4. Interpretation

In this stage, according to goal and scope of this study, the overall findings of inventory analysis and impact assessment are combined and discussed to gain conclusions and recommendations [19,20]. This is a systematic technique to check and evaluate information from the results of the LCI and LCIA, and communicate them effectively. Indeed, the interpretation stage is based on the contribution check of the quantified hotspots and identified elements (input, processes and stages) which will have the greatest influence on certain impact category or on the total life cycle impacts.

#### Results Evaluation

The interpretation includes a critical reflection about the study, through sensitivity check and/or uncertainty analysis related to representativeness of input data and adequacy of methodological choices. In this study, to test the robustness of the LCA results and assess the influence of the key parameters on the evaluated impact categories, both an uncertainty analysis and a sensitivity check were carried out.

The uncertainty analysis was performed by the Monte Carlo (MC) functional unit within SimaPro 8.0.3 software, in order to propagate the uncertainty linked to foreground input/output of the crop stage (see Section 2.2.1) and industrial phase (see Section 2.2.2). For background inputs and emissions of both feedstock cultivation and processing, the probability distributions were retrieved from the EcoInvent v. 2.0 database. The 95% confidence limits were generated by running simulations of 10,000 trials.

On the basis of the main outcomes of the LCA analysis, the sensitivity check was used to evaluate and argue the potential implementation of emerged key steps, through possible alternative routes along the bio-based BDO production chain.

## 3. Results

### 3.1. Cradle-to-Factory Gate Impacts of Bio-Based BDO

**Figure 2:**
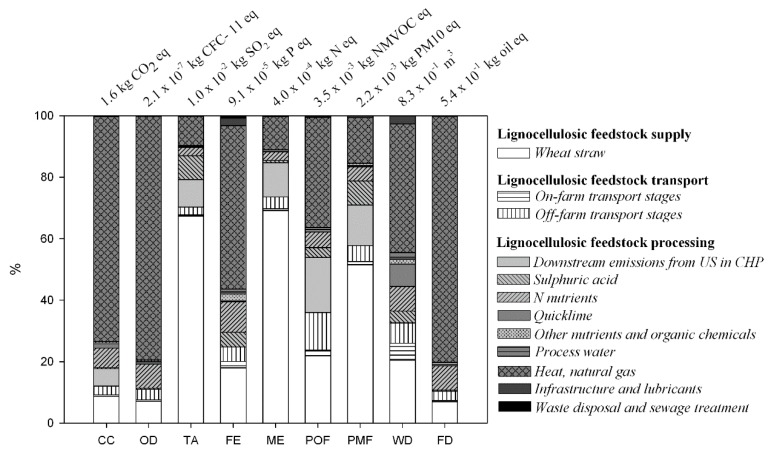
Characterization graph related to the production of 1 kg of butane-1,4-diol bio. Absolute values of impacts are reported on the top of each impact category. US: unconverted solids; CHP: combined heat and power system.

Figure 2 shows, for each selected impact category, the total impact and the different share of the several input and output along the main stages of the BDO production chain. 

The supply of the lignocellulosic feedstock and the extra-input heat (not obtained by US combustion in the internal CHP) appeared the prevailing process stages affecting the total environmental impact (Figure 2). Natural gas input determined the greatest share for FD and CC, due to downstream fossil CO_2_ emissions during gas burning. Specifically it represented the key relevant industrial input, thanks to the use of carbon-neutral renewable lignocellulosic feedstock (Table 4).

The additional heat supply at the bio-refinery plant also affected OD, FE and WD, as a consequence of upstream emissions (bromochlorodifluoromethane and phosphate) and input (water) related to the gas supply chain, mainly linked to the electricity requirement. POF was driven to similar extents by upstream (i.e., from agricultural machinery and natural gas supply) and downstream (during agricultural machinery operations in the field and combustion at the CHP) NO_x_ emissions. The impact of WS arose markedly (up to about 90%) as far as TA, ME and PMF were concerned, due to the N fertilization.

The impact of others input/output, along the biomass industrial processing, appeared limited (Figure 2). Nonetheless, N nutrients (involved within the saccharification and the fermentation steps) contributed to about 10% of total impacts, as averaged value for all impact categories. The share of sulphuric acid (at the pre-treatment stage) emerged in terms of TA and PMF, due to upstream sulphur dioxide emissions linked to the secondary sulphur input and air pollutant emissions at chemical plant; whilst tailpipe emissions of NO_x_ and non-methane organic volatile compounds lead to the highest share of transport stages to POF.

#### Cradle-to-Farm Gate Impacts of Lignocellulosic Feedstock Production

As indicated in the goal and scope section, the present study has conceived the wheat cultivation to obtain the two co-products to be sold on the market, wheat grains and wheat straw, the latter to be used into the investigated hypothetical regional bio-refinery chain.

**Figure 3:**
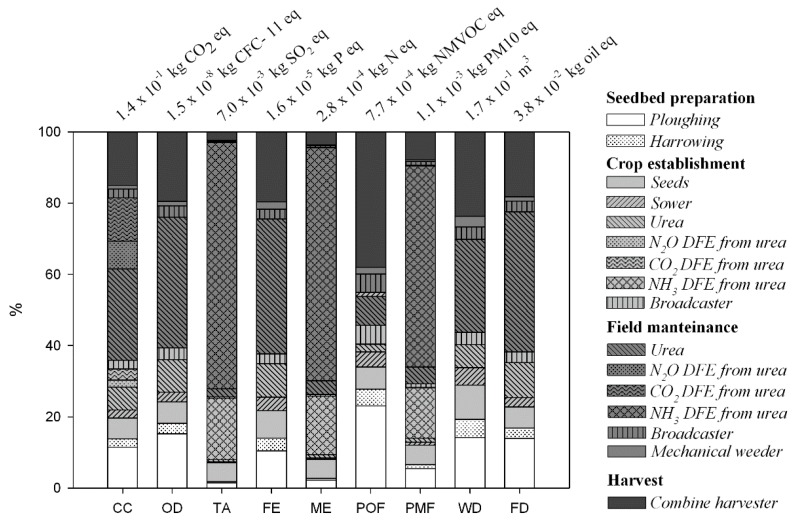
Characterization graph of impacts along the WS feedstock agronomic supply chain, related to the production of 1 kg of butane-1,4-diol bio. Absolute values of impacts are reported on top of each impact category. DFE: direct field emissions from the cropped soil.

Focusing on the agronomic stage of the WS feedstock cultivation, the N fertilization carried out at crop establishment and later in spring (for field maintenance) emerged as the prevailing contributor to almost all the impact categories (Figure 3). Additionally, according to the scheduling of N fertilization along the wheat cultivation in Campania Region (Table 1), the impacts arose moving from the sowing to the late fertilization, characterized by a higher application rate (Figure 3). 

As it relates to the specific impacts, OD, FE, WD and FD were affected by upstream emissions (halocarbures, phosphate) and input (turbine water and fossil energy mix) mainly linked to the urea production. For CC, TA, ME and PMF there was a relevant share from DFE of greenhouse gases (GHG) and reactive N caused by the N fertilizer supply (Figure 3). Specifically, CO_2_ emissions (fossil, from urea molecule decomposition in soil) and N_2_O biogenic fluxes from soil amounted to about 15% and 10% of the total CC impact, respectively (as a sum of emissions at sowing and late fertilizations). DFE relevance arose for TA, ME and PMF, for which about 92% of total WS environmental load was shared by the sowing and the late fertilization events (24% and 68%, respectively) (Figure 3). This result was almost entirely related to the volatilized NH_3_ following urea application at sowing and field maintenance (about 70% and 90%, respectively, as average values for TA, ME and PMF).

Differently, POF was driven by the pattern of fuel consumption (and linked exhaust emissions) along the different agronomic practice and therefore resulted mainly affected by the higher diesel input required for soil preparation and harvest (Figure 3).

### 3.2. Bio-Based BDO vs. the Fossil Counterpart

**Figure 4:**
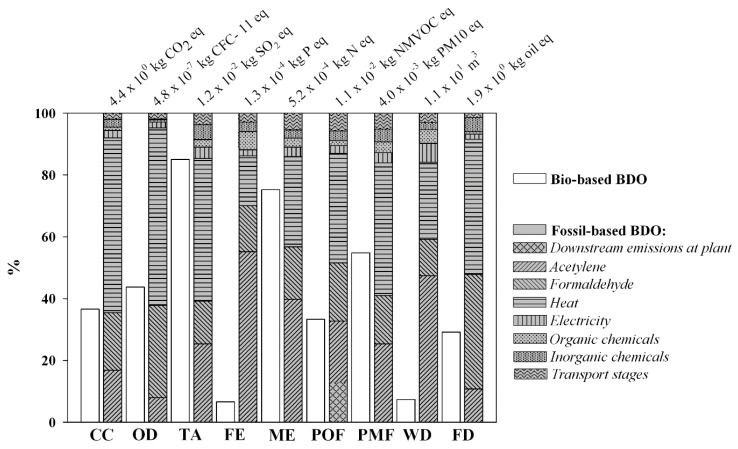
The characterization graph shows the comparison between 1 kg of bio-based BDO and 1 kg of fossil-based BDO. The figure also details the contribution from the different input process to the total impacts of the fossil-based BDO production (“butane-1,4-diol, at plant” record, from EcoInvent database v. 2.02).

In Figure 4, the environmental impact of BDO-bio was compared to the BDO-fossil on the basis of similar system boundary and functional unit (see Section 2.1).

The environmental load of the bio-based BDO resulted consistently lower as compared to fossil BDO, whose impacts appeared driven by the fossil (coal, oil and natural gas) energy sources (for CC, OD, TA and FD) and by the inputs of formaldehyde and acetylene (mainly for FE, ME and WD). These latter ones are produced through emission intensive industrial processes, respectively, greatly relying on: (i) natural gas supply, as common raw material and energy source in the catalytic reforming step to create synthesis gas for methanol production (formaldehyde); and (ii) electricity input for liquid oxygen production (acetylene).

Respect to the conventional fossil supply chain, the bio-based system relayed on a renewable C source as raw material for the production of BDO (0.5 kg of biogenic C incorporated in each kg of produced BDO) (Table 4). At the same time, the US co-produced in the conversion steps (Figure 1) were assumed to be burned in an internal CHP plant, for the co-generation of electricity and heat to be used inside the biorefinery system. This resulted in lower net energy requirement as opposite to the fossil based BDO. Moreover, the downstream C pollutant air emissions at the CHP plant were accounted as biogenic, and specifically were assumed neutral as far as the C–CO_2_ was concerned (about 5 kg biogenic CO_2_ per kg BDO produced) (Table 4).

This led to markedly lower impacts of the bio-based BDO in terms of CC, OD, POF and FD (by about 65% as averaged value) (Figure 4). Higher benefits were gained even for FE and WD, due to the relevant load of fossil BDO related to: (i) upstream phosphate emissions and water input (for turbine use) connected to the electricity input along the natural gas; and (ii) the liquid oxygen supply chain for the production of formaldehyde and acetylene. On the other hand, for those impact categories affected by the WS feedstock cultivation (TA, ME and PMF), discrepancies between the bio- and the fossil- based BDO appeared more circumscribed, especially for TA, driven by ammonia emissions following the N fertilization practice (Figure 3).

### 3.3. Results Evaluation

#### 3.3.1. Uncertainty Analysis

**Figure 5:**
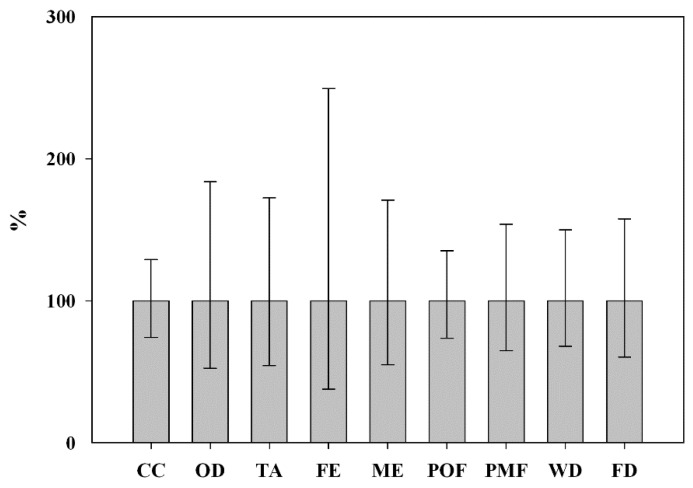
Graphical representation of confidence interval (95%) for impacts related to the overall production chain of 1 kg of bio-based BDO.

Impacts assessed of the bio-based BDO were affected by uncertainties, related to the whole production chain (Figure 5).

On the whole, observed variance mostly came from the uncertainty linked to the foreground input of additional heat and lignocellulosic feedstock conversion at the biorefinery plant, in their turn affected by the range of BDO recovery efficiency through C5 and C6 sugars co-fermentation.

Specifically (Table 5), uncertainty of CC, OD, FE, POF, WD and FD appeared driven by the amount of natural gas required at the biorefinery plant and by its link to: (i) upstream input of water (in turbine) and emissions of halocarbons, phosphate and nitrogen oxides; and (ii) downstream emissions of fossil CO_2_ and NO_x_ during gas combustion in furnace. As it relates to FE, the wider variability range was also determined by the uncertainty of background input (and linked upstream emissions) related to both the agronomic and industrial stages (i.e., the production of agricultural fertilizers, machineries, etc.). Differently the 95% confidence interval of TA, ME and PMF was largely affected by the assumption inherent to the agronomic yield and the type and the number of needed practices along the feedstock cultivation. Specifically the uncertainty appeared linked to representativeness of foreground DFE of ammonia at sowing and late fertilization (Table 5). The potential nitrates in groundwater did not represent a significant driver of ME variance. This following the negligible risk of leaching under Mediterranean conditions at a fertilization rate <200 kg·N·ha^−1^ split between sowing and stem elongation [42,45], which was applied as BG and LB for the uncertainty range of N–NO_3_^−^ losses. Likewise, the possibility of rescue irrigation water (with respect to the rainfed cultivation applied as BG and LB in the study) did not substantially affect uncertainty of WD at the crop phase, which appeared mainly affected by the variance of up-stream water input along the supply chains of agricultural machineries and other agronomic auxiliaries.

Evaluating the uncertainties related to the whole production chains, the environmental benefits of the bio-based BDO would be confirmed in terms of CC, FE, POF, WD and FD mitigation. They might be not significant for TA, ME and PMF.

#### 3.3.2. Sensitivity Check

As highlighted by the main results of the cradle-to-factory gate LCA and the uncertainty analysis, two key aspects can be implemented in order to tune the final outcome of the LCA towards an higher environmental performance: (i) efficiency of feedstock conversion, which in its turn affects the level of WS feedstock input and linked impacts from the agronomic cultivation stage on target impact categories; (ii) extra heat supplied, not covered by the combustion of US in the CHP plant.

**Figure 6:**
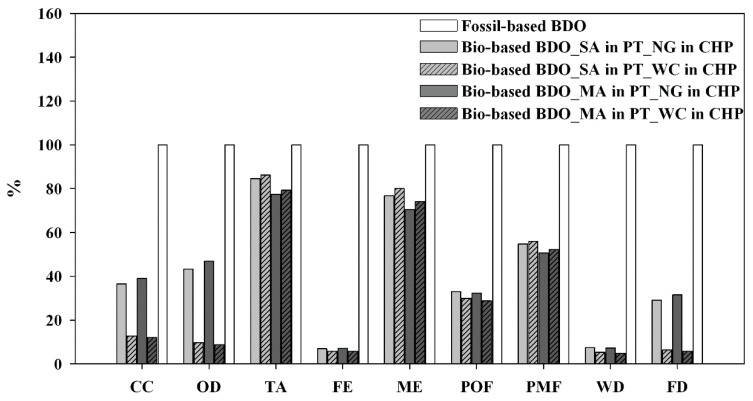
The characterization graph shows the relative impacts of the bio-based BDO, along the different routes subject to the sensitivity analysis, respect to the fossil-based BDO. Data referred to 1 kg of BDO production. SA in PT: sulfuric acid in pre-treatment; NG in CHP: additional heat supplied by natural gas in combined heat and power co-generator; MA in PT: maleic acid in pre-treatment; WC in CHP: additional heat supplied by wood chips in combined heat and power co-generator.

Therefore, a preliminary sensitivity check (Figure 6) was performed to analyze the potential improvements of the investigated bio-based supply chain in the case of: (i) dilute acid pre-treatment through maleic acid (instead of sulphuric acid) coupled with enzymatic hydrolysis, which showed to implement sugars recovery efficiency from WS feedstock to 85% of glucose and 80% of xylose, thanks to the reduced sugar degradation reactions [53]; (ii) additional heat supply through the co-combustion of wood chips in CHP plant, as renewable alternative to natural gas.

The replacement of sulfuric acid with maleic acid within the pre-treatment step led to increased yield of bio-based BDO. At the same time it increased the additional external energy input to counteract the reduced US mass flow to CHP plant. As a result, impacts appeared reduced for those categories sensitive to the environmental load of the feedstock cultivation (TA, ME and PMF); whilst they appeared invariant or slightly increased for categories driven by the on-site generation of heat through natural gas (Figure 6).

Conversely, under the configuration of extra heat supply by wood chips combustion in CHP plant, the sensitivity analysis highlighted a marked improvement of the bio-based BDO performance in terms of CC, OD and FD, coupled to increased impacts in terms of TA, ME and PMF, due to the wood chips cultivation stage.

According to these preliminary results, the most promising compromise configuration would be the combination of dilute organic acid pre-treatment with wood chips burning in CHP plant.

## 4. Discussion

### 4.1. Cradle-to-Factory Gate Impacts of Bio-Based BDO

The environmental load of the bio-based BDO resulted mainly affected by two stages: (i) heat energetic supply at the biorefinery plant, which is also a key factor for the environmental profile of the fossil-based BDO (EcoInvent v. 2.0 database) [37]; (ii) the agronomic stage of feedstock cultivation, which ranged from about 10% to about 70% of total impacts related to the whole production chain (Figure 2). The latter result inserts itself in the wider context of bioethanol, bioenergy, and biochemicalsbiorefinery platforms from lignocellulosic feedstock, for which the crop phase appeared significant [58,59,60,61].

Specifically, volatilized ammonia played a key role entailing about 60%, 56% and 36% of total TA, ME and PMF impacts, respectively. Field emissions of reactive nitrogen are recognized as a high source of uncertainty in relation to the impacts of the cultivation stage along biomass supply chains [62]. They have been highlighted as relevant responsible of acidification and eutrophication impacts related to the biomass feedstock cultivation stage [46] and to total production chains from biomass feedstock towards fermentable sugars [63], steam [64] and bioethanol [65]. GHG DFE, which also appeared significant along the crop phase and represent a recognized contributor to the GWP of bio-based production chains [40,53,54,56,57], accounted only for about 2% of the total CC impact, mostly driven by the heat supply at the biorefinery plant. As far as biogenic N_2_O fluxes were concerned, this result was also affected by the local EF applied in the present study to better represent soil bacterial GHG emissions under Mediterranean conditions [46]. By the use of the IPCC default EF [48], N_2_O biogenic contribution would have scored up to about 5% of total CC.

Withregards to the impacts from the extra heat sources supply, the substitution of fossil energy with other form of renewable energy may lead to general improvements of the bio-based BDO environmental performance. However, the topic needs to be carefully addressed. As highlighted by the sensitivity analysis (Section 3.3.2), the replacement with pellets might increase impacts in terms of acidification and eutrophication, which are often already hindered impact categories when comparing bio-compounds (bio-fuels, bio-polymers) with the respective fossil counterparts (see Section 4.2 for further details).

### 4.2. Potential Benefits of Bio-Based BDO vs. the Fossil Counterpart

Industrial bio-based technologies can offer process pathways with lower carbon footprint than petrochemicals. Additionally, they are also often cleaner than fossil reference routes. This thanks to the recovery and valorization of co/by products within the integrated biorefinery, which lead to reduced net energy consumption and wastes (i.e., combustion of residues for on-site energy production) as opposite to conventional synthetic processes [11,66,67]. The present case study highlighted a general reduction of impacts of the bio-based BDO respect to the fossil counterpart.

The climate and energy mitigation potential appeared in line with results inherent to the production of compounds from biological sources [16,18,28]. Specifically, according to Adom et al. [16], cradle-to-grave GHG emission of bio-based 1,4 BDO produced from corn stover-derived sugars resulted about 52% lower respect to the fossil-based counterpart. Cok et al. [22] found that succinic acid produced from corn-derived dextrose (through fermentation and crystallization) has a lower (by 67% to 92%) life-cycle GHG intensity than fossil-derived maleic, adipic, and succinic acids. Similarly, Urban and Bakshi [28] concluded that the bio-based 1,3propanediol from corn was 46%–71% less GHG-intensive than the fossil counterpart, depending on the different system boundaries encompassed in their life-cycle evaluation. As far as the non-renewable energy consumption, the study highlighted for bio-based BDO reduced total impact in terms of FD by 72% respect to fossil-based BDO. According to Groot et al. [68], this was the result of the reductionof: (i) feedstock related impacts (by about 94%, respect to fossil-based BDO), thanks to the substitution of acetylene and formaldehyde with the renewable wheat straw C feedstock; (ii) process related impacts (by about 50%, respect to fossil-based BDO). With regard to the second point, the modeled bio-based supply chain required less net non-renewable foreground energy input for feedstock processing at the industrial plant (about −40%), thanks to the combustion of unconverted biomass residues in the cogeneration unit, as usually highlighted for advanced integrated systems of biorefinery [69,70,71].

However most studies focused on the climate change and fossil depletion impact; whilst the whole environmental performance of bio-based products has been less investigated. In this context, some contrasting findings arose. Among four tested bio-chemicals from sugar beet vinasse, only one bio-based compound (pyrrolidone) showed a whole better environmental profile as opposite to the petroleum counterparts [29]. Conversely to the identified benefits in terms of decreased fossil fuel use and global warming potential, the life-cycle evaluation of bio-polymers has shown increased environmental load in impact categories such as eutrophication [18,28] and human health impacts and eco-toxicity [18]. Additionally, they could be responsible of a larger general stress on ecosystems in terms of land use, water use, soil erosion, and nutrient runoff [28]. In the present study, benefits of the bio-based BDO resulted more circumscribed for those impact categories highly affected by fertilization along the feedstock cultivation stage. This appeared in line with the wider general results for bio-fuels production chains, which exhibited acidification and eutrophication impacts comparable (even higher) respect to conventional fossil fuels, due to the cultivation stage [71,72].

Therefore, some preliminary consideration could be formulated towards the improvements of the environmental performance of the bio-based BDO, encompassing different steps of the whole production chain.

- Agronomic practices: The N fertilizer management could be implemented by optimizing fertilization rates and/or through the use of N formulations with reduced potential for NH_3_ volatilization, such as ammonium nitrate fertilizers [41] or urea-containing fertilizers with urease inhibitors to constrain the downstream emissions of NH_3_ [73,74,75]. However different rate and formulations of N fertilizer might result in different agronomic yields and costs of fertilization practice, which also should be taken in consideration. Similarly, the effective mitigation potential of inhibitors in terms of ammonia volatilization need to be further verified [76].
- Methodological assumptions: Site-specific NH_3_ volatilization emission and characterization factors would be beneficial for more reliable estimate of NH_3_ emissions from Mediterranean cropped soils, which appear not yet extensively investigated [46].
- Technical aspects inherent the industrial processing steps: Higher holocelluloses recovery efficiencies, as well as fermentation yields, would affect and restrain indirectly the environmental load of the crop phase. Considering all this, new analyses will be performed to test the benefits of other potential pathways for biomass processing (such as steam-explosion pre-treatment) and identify the most environmental promising routes to achieve scalable integrated biorefinery chains for BDO production.

### 4.3. Concerns about WS Feedstock Availability and Potential Competition with Current Use

Considering the wheat straw availability [38] and the efficiencies of sugars recovery and fermentation applied in this study, the hypothetical local biorefinery system might produce about 12 kton per year of bio-based BDO, which might be competitive with capacities of developing and existing BDO facilities [14]. Results appeared to confirm the potential of wheat straw feedstock for further effective processing within regional bio-based production chains, which has recently been highlighted in the European context [77,78]. A remarkable matter in this regard (industry logistics) is the “potential availability” of straw biomass depending on climatic and agronomic factors such as cereal varieties, straw height at harvest, straw-to-grain ratio, etc. [78]. Nevertheless, the “real-availability” of straw lignocellulosic feedstock is driven by other critical points, usually identified as follow: (i) lack of detailed information about total amount and existing utilization routes of cereal stubble [78,79], which prevents from a reliable understanding of the feasibility of stalk-based production chains within the framework of the current market; (ii) potential competition with the common practice of soil incorporation, which entails benefits in term of preservation of both soil organic carbon (SOC) and nutrients [78,79,80,81,82,83].

According to recent studies, the effect of straw removal on SOC is still controversial [81,82,83]. This is mainly due to the mediation effect of plant roots and microbial biomass, in their turn affected by the water input management [81,82]. However it is possible to derive a tentative estimate of the minimum quantity of above-ground wheat residue that needs to be left in the field to maintain SOC levels at different grain yields [82]. As it relates to the nutrient losses caused by stubble removal, the framework is even more composite. On one hand, it is commonly accepted that the removal of straw, rich in potash and phosphate, lead to additional input of P_2_O_5_ and K_2_O synthetic fertilizers [79,81,82,83]. On the other one, the long term effect in term of N depletion and fertilizer requirement is less certain [79], whilst in the short term extra N chemical fertilizer input is usually required to counterbalance the temporary ‘lock up’ of N during the microbial decomposition of biomass residues [39,79,81,82]. Therefore, it is not possible to place a simplified substitution factor for N removed in straw [79,81,82].

This study assumed as potential lignocellulosic feedstock only the available fraction of wheat straw, outside (and therefore not in competition with) its stated current agronomic and zootechnical use [38]. However updated estimates and more detailed information about the current fate of this “surplus” cereal straw within the regional context would be beneficial. Similarly it would be useful to get disaggregated data about the different use pathways of straw (on farm and off farm use for animal feed and bedding, soil incorporation, energy production by combustion in combined heat and power cogeneration systems, etc.), which could not be retrieved from the available statistics [38]. In this way, it could be possible to further detail and encompass inside future upgraded LCA analyses of bio-based BDO production in the regional context: (i) the maximum amount of wheat residue that could be harvested, to maintain SOC under regional cereal crops; (ii) the eventual additional environmental and economic loads (extra synthetic and organic fertilizers/conditioners) to return P-K nutrients and organic matter back into the soil (i.e., to determine the agronomic value of the straw).

This information could support policy decision processes about straw fate (whether remove or incorporate) under new developing biorenewable markets. Indeed, currently, the rate of stubble incorporation in soil is affected also by the relevant cost of straw collection, baling and transport operations, which account for a huge fraction of the delivered value of straw feedstock [78,79,80]. However, these perceived economic problems of straw removal might be scaled down in the next future, when straw sales might provide a valuable revenue stream along emerging bio-based production chains and therefore drive straw utilization patterns.

The biorefinery concept is a quite complex system and a reliable assessment of the most promising bio-based production pathways need to encompass all the technical barriers as well as wider sustainability environmental, economic and social issues [11,21]. Even if the bio-based products are generally recognized as less energy and GHG emissions intensive [16,22,28], the current trend of converting traditional fossil-based products to bio-based should be explored and proceed with caution. Coupling the increased interest in producing bio-products with the large production of biofuel from biomass (even though not in competition with food supply chain), a scenario in which the feedstock biomass become scarce is not farfetched. From a decision making point of view, GHG emissions and fossil fuel consumption will be meaningless quantities if there are not enough natural resources to go around. For this reason it is extremely important to address the sustainability issue taking into account depletion of the natural resources and the increase deteriorating state of natural ecosystem.

## 5. Conclusions

This paper represents a first piece in the complex mosaic of studying the feasibility of a territorial biorefinery toward the sustainable principles.

It has dealt the environmental performance of BDO production chain, with main goals (i) individuate the hot spots production stages; and (ii) assess potential benefits compared with fossil counterpart. According to the results achieved in this LCA study, hot spots have been identified in the energy demand of the entire process and in the feedstock production. Our assessment pointed out that the available wheat straw lignocellulosic feedstock in Campania Region (not in competition with current agro-zootechnical use) appeared as a suitable alternative source for eco-friendly bio-based BDO production within a hypothetical local biorefinery scenario. 

Several issues need to be further addressed to: (i) constrain the impact of lignocellulosic feedstock cultivation; (ii) implement technical aspects of the industrial bio-based manufacturing process; and (iii) achieve in the future a proper balance between agricultural and industrial use of cereal stalks. As far as the last point is concerned, updated large-scale surveys appear necessary to characterize local straw availability and current destination routes. This in order to assess the viability of straw market as a renewable material, respecting its agronomic use through soil incorporation and its linked benefits in terms of enhancement of soil nutrient and physical properties. Additionally, further topics need to be encompassed inherent to economic aspects such as policy incentives and directives, which were behind the scope of the present work, but can heavily affect commercial farming practices and market dynamics. Indeed, the issue of sustainable is complex and requires robust and integrated scientific studies in order to address accurately policy decisions, stakeholders’ interests and social needs.

## Figures and Tables

**Table 1:** Best guess (BG) for agronomic input and output of wheat cultivation. Lower and upper bounds (LB and UB, respectively) of uncertainty range and type of applied distribution (D) are also reported.

Agronomic Input/Output ^a^			Unit	BG	Uncertainity Range		
					LB	UB	D
Input ^a^	Soil preparation	Tillage, ploughing	n.·ha^−1^	1	0	1	Triangular
		Tillage, harrowing	n.·ha^−1^	1	0	2	Triangular
	Sowing	Wheat seeds	kg·ha^−1^	190	87	285	Triangular
		Sowing	n.·ha^−1^	1	–	–	–
		Triple superphosphate, as P_2_O_5_	kg·ha^−1^	0	0	70	Triangular
		Potassium sulphate, as K_2_O	kg·ha^−1^	0	0	70	Triangular
		Urea, as N	kg·ha^−1^	20	0	26	Triangular
		Fertilizing, by broadcaster	n.·ha^−1^	1	0	1	Triangular
	Field maintenance	Urea, as N	kg·ha^−1^	80	60	104	Triangular
		Fertilizing, by broadcaster	n.·ha^−1^	1	1	2	Triangular
		Tillage, currying, by weeder	n.·ha^−1^	1	0	2	Triangular
		Pesticide	kg·ha^−1^	0	0	1.6	Triangular
		Application of pesticide	n.·ha^−1^	0	0	2	Triangular
		Irrigation water	m^3^·ha^−1^	0	0	400	Triangular
	Harvest	Combine harvesting	n.·ha^−1^	1	–	–	–
Output	Yields	Grain ^b^	ton·ha^−1^	3.1	2.1	6.2	Uniform
		Straw ^c^	ton·ha^−1^	5.6	3.8	11.2	Uniform
	Agronomic DFE ^d^	NH_3_, volatilized	kg·ha^−1^	18.2	7.4	31.6	Uniform
		N_2_O, biogenic	kg·ha^−1^	0.4	0.2	2.8	Uniform
		NO_3_ leached	kg·ha^−1^	0	0	274.6	Uniform
		CO_2_ fossil from N-urea	kg·ha^−1^	157	94.2	204.1	Uniform
		PO_4_^3−^ runoff to surface water	kg·ha^−1^	0	0	0.5	Uniform
		PO_4_^3−^ leaching to ground water	kg·ha^−1^	0	0	0.2	Uniform
		P runoff to surface water	kg·ha^−1^	0	0	0.1	Uniform

^a^ [39,42,43,45,50]; ^b^ Grain yield values retrieved from [51]; ^c^ Straw yield values based on an averaged straw-to-grain ratio of about 2 [38,42,44]; ^d^ Lower and upper bounds of DFE were derived on the basis of the combined effect of fertilizers input variations and uncertainty ranges of emission factors: 10%–20% for NH_3_–N volatilization factor, 0.08%–1% for N_2_O–N emission factor and 0.79–1.57 for kg CO_2_ fossil emissions from 1 kg applied urea-N [46,47,48]; 0–5 ton·year^−1^ as range of soil erosion from regional plain cropped soil [49].

**Table 2:** Data input/output for 1 kg of bio-based BDO, related to the whole industrial phase: dilute sulphuric acid pre-treatment, enzymatic hydrolysis, C5andC6 co-fermentation and BDO purification.

Industrial Input/Output		Amount	Unit Measure
**Input**	Water ^a^	5.8	kg·kg^−1^_BDO_
	Sulphuric acid	0.06	kg·kg^−1^_BDO_
	Nutrients and organic chemicals ^b^	0.3	kg·kg^−1^_BDO_
	Quicklime	0.05	kg·kg^−1^_BDO_
	Total energy consumption^c^	41	MJ·kg^−1^_BDO_
**Output**	BDO-bio	1	kg

^a^ Net total amount, recycling included; ^b^ Sum of nutrients and organic chemicals linked to enzymatic hydrolysis and co-fermentation (i.e., ammonium sulphate, liquid ammonia, magnesium sulphate, etc.); ^c^ Sum of total electricity and heat required. The plant appeared self-sufficient (through combustion of unconverted solid in CHP) for EE input; whilst an additional amount of 17 MJ of heat per kg BDO was required and assumed supplied to the system by natural gas burning.

**Table 3:** Characteristics of unconverted solids. Best guess (BG), Lower and Upper bounds (LB and UB, respectively) and type of applied distribution (D) are reported.

Parameter	Unit	BG	Uncertainty Range		
			LB	UB	D
Dry matter input ^a^	kg·kg^−1^_ws(db)_	0.52	0.42	0.56	Uniform
Carbon input, biogenic ^a^	kg·kg^−1^_ws(db)_	0.28	0.24	0.32	Uniform
Energy input ^b^	MJ·kg^−1^_US_	8.9	8.7	9.5	Uniform
Heat production ^c^	MJ·kg^−1^_BDO_	20	13	35	Uniform
Electricity production ^d^	kWh·kg^−1^_BDO_	2	1	2	Uniform

^a^ According to biomass and C biogenic flows along the industrial processing steps within the three different fermentation efficiency routes, assuming an average C content in WS of 0.44 [56]; WS (db): wheat straw, dry basis; ^b^ Gross calorific values for cellulose, hemicellulose and sugars derived from literature were converted to LHV through the Hartmann equation, assuming about 55% water content (% *w*/*w*) in US; ^c^ Assuming heat losses coefficients of 20% for boiler and 5% for heat exchanger; ^d^ Assuming efficiency of EE production close to 18%.

**Table 4:** C biogenic balance related to 1 kg of bio-based BDO production.

C Biogenic	KgCkg^−1^ BDO
Input ^1^	–
C in WS feedstock	2
Output ^1^	–
C in bio-based BDO	0.5
C in downstream emissions from industrial processing of WS feedstock ^2^:	–
*Co-fermentation*	–
C–CO_2_	0.3
*US combustion in CHP*	–
C–CH_4_^1^	6 × 10^−6^
C–CO ^1^	6 × 10^−5^
*C-*non methan organic compounds ^2^	8 × 10^−5^
C–CO_2_^1^	1.2
Total output C	2

^1^ General note to the table. The C balance was computed on the basis of the assumed averaged WS feedstock composition [44,45,46] and the C content [56], according to the biomass flows along the designed industrial processing in compliance with: (i) sugars recovery efficiency (74%) after dilute sulphuric acid pre-treatment and enzymatic hydrolysis from Volynets and Dahman [53]; (ii) co-fermentation and purification yield (experimental primary data within the framework of the “EnerBiochem” Project). The downstream C emissions during combustion of US in CHP plant were shared among the different C-compounds according to the EcoInventrecord “wood chips, burned in cogen 6400 kWth, emission control”, properly adapted on the basis of dry matter, carbon and energy content of the analyzed US, following EcoInvent guidelines (EcoInvent Report n. 17). WS: wheat straw; US: unconverted solids; CHP: combined heat and power system.

**Table 5:** Results from the Monte Carlo Uncertainty analysis related to 1 kg of the bio-based BDO production, for key input processes and linked pollutant emissions. Ds: downstream; Us: upstream; DFE: direct field emissions.

Impact Category	Total Impact				Impact from Key Input Process				Input/Output	Impact from Key Pollutant Input/Output			
	Mean ^a^	SD ^b^	CI ^c^		Mean	SD ^b^	CI ^c^			Mean	SD ^b^	CI ^c^	
			2.5%	97.5%			2.5%	97.5%				2.5%	97.5%
					Additional Heat					Key Input/Output from Additional Heat			
CC (kg CO2 eq)	1.6 × 10^0^	2.4 × 10^−1^	1.2 × 10^0^	2.1 × 10^0^	1.1 × 10^0^	2.2 × 10^−1^	7.6 × 10^−1^	1.6 × 10^0^	CO_2_ (Ds, fossil)	1.1 × 10^0^	2.0 × 10^−1^	7.2 × 10^−1^	1.5 × 10^0^
OD (kg CFC-11 eq)	2.0 × 10^−7^	7.0 × 10^−8^	1.1 × 10^−7^	3.8 × 10^−7^	1.6 × 10^−7^	6.5 × 10^−8^	7.3 × 10^−8^	3.2 × 10^−7^	Halon 1211 (Us)	1.5 × 10^−7^	6.4 × 10^−8^	6.9 × 10^−8^	3.1 × 10^−7^
FE (kg P eq)	9.2 × 10^−5^	5.5 × 10^−5^	3.5 × 10^−5^	2.3 × 10^−4^	4.7 × 10^−5^	3.6 × 10^−5^	1.2 × 10^−5^	1.4 × 10^−4^	Phosphate (Us)	4.7 × 10^−5^	3.6 × 10^−5^	1.2 × 10^−5^	1.4 × 10^−4^
POF (kg NMVOC eq)	3.6 × 10^−3^	5.7 × 10^−4^	2.6 × 10^−3^	4.9 × 10^−3^	1.2 × 10^−3^	4.0 × 10^−4^	6.3 × 10^−4^	2.2 × 10^−3^	NO*_×_* (Us/Ds)	8.1 × 10^−4^	3.2 × 10^−4^	3.7 × 10^−4^	1.6 × 10^−3^
WD (m^3^)	8.4 × 10^−1^	1.7 × 10^−1^	5.7 × 10^−1^	1.3 × 10^0^	3.3 × 10^−1^	1.4 × 10^−1^	1.5 × 10^−1^	6.7 × 10^−1^	Water (Us, turbin)	3.3 × 10^−1^	1.4 × 10^−1^	1.5 × 10^−1^	6.7 × 10^−1^
FD (kg oil eq)	5.3 × 10^−1^	1.4 × 10^−1^	3.2 × 10^−1^	8.4 × 10^−1^	4.2 × 10^−1^	1.3 × 10^−1^	2.3 × 10^−1^	7.1 × 10^−1^	Natural gas (Us)	4.1 × 10^−1^	1.2 × 10^−1^	2.2 × 10^−1^	7.0 × 10^−1^
					**Wheat Straw**				**Input/Output**	**Key Output from Wheat Straw**			
TA (kg SO2 eq)	1.2 × 10^−2^	3.8 × 10^−3^	6.7 × 10^−3^	2.1 × 10^−2^	8.8 × 10^−3^	3.6 × 10^−3^	3.4 × 10^−3^	1.7 × 10^−2^	NH_3_ (DFE)	8.2 × 10^−3^	3.5 × 10^−3^	3.0 × 10^−3^	1.7 × 10^−2^
ME (kg N eq)	4.8 × 10^−4^	1.4 × 10^−4^	2.6 × 10^−4^	8.1 × 10^−4^	3.5 × 10^−4^	1.4 × 10^−4^	1.4 × 10^−4^	6.8 × 10^−4^	NH_3_ (DFE)	3.1 × 10^−4^	1.3 × 10^−4^	1.1 × 10^−4^	6.2 × 10^−4^
PMF (kg PM10 eq)	2.4 × 10^−3^	5.6 × 10^−4^	1.6 × 10^−3^	3.8 × 10^−3^	1.4 × 10^−3^	5.1 × 10^−4^	6.1 × 10^−4^	2.6 × 10^−3^	NH_3_ (DFE)	1.1 × 10^−3^	4.6 × 10^−4^	3.9 × 10^−4^	2.2 × 10^−3^

^a^ Similar values for mean and median; ^b^ Standard deviation; ^c^ Confidence Interval.

## Abbreviations

The following abbreviations are used in this manuscript:

CC	Climate change;
OD	Ozone depletion;
TA	Terrestrial acidification;
FE	Freshwater eutrophication;
ME	Marine eutrophication;
POF	Photochemical oxidant formation;
PMF	Particulate matter formation;
WD	Water depletion;
FD	Fossil depletion;
SOC	Soil organic carbon;
WS	Wheat straw;
US	Uncorverted solids;
CHP	Combined Heat and Power.
